# A Fatal Switch for Corals?

**DOI:** 10.1371/journal.pbio.1000346

**Published:** 2010-03-30

**Authors:** Elizabeth Whiteman

**Affiliations:** Consulting Editor, PLoS Biology, Public Library of Science, San Francisco, California, United States of America

In the late summer of 2005, abnormally warm ocean temperatures led to the most severe coral bleaching event ever documented in the northeast Caribbean. Algae living in symbiotic harmony with their animal hosts were expelled, leaving great expanses of white, or so-called bleached, coral reef. In the 12 months following these high temperatures, disease incidence on corals rose sharply. Within two years, more than 50% of live coral cover around islands, including the US Virgin Islands, would be gone, claimed in large part by disease.

These events, in which high ocean temperatures cause coral bleaching and subsequent coral death, appear to be occurring ever more frequently. The 2005 Caribbean die-off followed closely on the heels of massive coral bleaching and disease events recorded in the Indian Ocean and Great Barrier Reef in 1998 and again in 2002, and also in the wider Caribbean in 1997 and 1998.

As they survey and report the consequences of increasing disease outbreaks, researchers are also trying to understand the relationships between temperature, bleaching, disease, and coral death. Recent studies have indicated that understanding what happens in the layer of mucus on the surface of corals is critical to understanding disease transmission. This mucus layer hosts a diverse community of microbes that, under normal environmental conditions, are essential in maintaining the “health” of the coral itself. Many of the resident microbes produce antibiotics that help ward off invading pathogens. But when corals are stressed—for example, by elevated temperatures—the community of microbes suddenly switches. Resident species decline as pathogens associated with coral diseases take their place.

Understanding what causes this sudden switch is essential to identifying the drivers and dynamics of coral disease, but the responsible factors have proven difficult to determine. Field studies have been hampered by the immense diversity of both beneficial and pathogenic microbial communities associated with different coral species and with different disease complexes.

Given the challenges inherent in field approaches, Justin Mao-Jones and colleagues from Cornell University turned to modeling in a new study in this issue of *PLoS Biology* to provide a theoretical explanation for this microbial community shift. Although many of the details of the specific resident and invading pathogenic microbes causing coral disease are unclear, a model can overcome these uncertainties by simulating tens of thousands of scenarios. Thus, rather than focusing on a specific coral and pathogen interaction, the authors could vary individual model parameters to explore general dynamics of the mucus microbial community that may apply to many different coral species and diseases.

Replicating many of the individual patterns seen previously in field studies of diseased corals, the authors began by building a general model that describes the interactions among microbes in a homogenous mucus layer. During colder months that are unfavorable to pathogens, the modeled beneficial bacteria are able to outcompete and exclude the pathogens. As temperatures warm during summer months, the growth rate of pathogens increases, giving them a benefit. By producing antibiotics, however, the beneficial bacteria are able to hold these pathogens in check.

But a short-term increase in temperature, such as the late summer ocean warming observed in the Caribbean in 2005, causes antibiotic activity to decline. The equilibrium previously seen in the model is suddenly lost, and the system jumps to a pathogen-dominated state.

While many field studies have provided this same result, the authors can dissect their model and provide an explanation for how this switch occurs. They suggest that control of pathogens via antibiotic activity is the key to pathogen exclusion. Moreover, the modeled system exhibits two stable states: one dominated by antibiotic-producing beneficial bacteria and one pathogen-dominated. Even slow increases in temperature result in sudden switches between these states.

Testing the generality of their results further, Mao-Jones and colleagues increased the complexity of their model. Despite appearances, the surface mucus layer on corals is not homogenous and is continually changing as new mucus is produced by the corals and older mucus is lost into the surrounding water. In this new model, the authors introduced a gradient in nutrient and microbe concentrations from the coral surface to the surrounding water, and they included the production and loss of mucus from the coral. This could lead to very different results. For example, spatial variability in the mucus could lead to stable coexistence of both beneficial and pathogenic bacteria. Pathogens could invade areas of the mucus with fewer beneficial bacteria and escape the effects of antibiotics.

Strikingly, this is not the case. The contingent exclusion of pathogens followed by a sudden switch to pathogen dominance still occurs in this new model. The authors examined the sensitivity of this analysis and result, varying individual inputs to see if the result is dependent on one particular model parameter. Despite increasing the growth rates of invading pathogens, then changing the rates at which beneficial and pathogenic bacteria can move through the mucus, the same qualitative result holds.

These results are also sobering. The authors' model also replicates the pattern observed many times across many different coral reefs in which coral diseases persist long after water temperatures have returned to normal. More than six months following the 2005 temperature increases in the northeastern Caribbean, apparently recovering elkhorn corals *Acropora palmata* in Florida still showed no sign of antibiotic activity in the surface mucus layer. Only when conditions become unusually unfavorable to pathogens can beneficial bacteria in the coral mucus layer return to dominance and play their role in protecting the coral from invading pathogens. In the northeast Caribbean in 2005, these circumstances arose too late for many corals.

Mao-Jones and colleagues address just one important interaction between beneficial and pathogenic bacteria in coral mucus. As with many such studies, the results provide insights into one aspect of pathogen invasion and also lead to many more questions. Why, for example, does antibiotic production decline as sea temperatures increase? How are these bacterial interactions in the mucus layer altered by an immune response from the coral itself? Future field and modeling studies will hopefully provide new insights into these questions.

But as they watch the development of the El Nino currently affecting the Pacific Ocean, researchers brace themselves for another potential summer of high ocean temperatures, coral bleaching, and disease. In the meantime, it's worth noting a broad practical implication of the authors' findings in this paper: preventing the sudden switch to pathogen dominance by tackling stressors, such as poor water quality, may still be easier than reversing the shift to pathogen dominance once it has occurred.

**Mao-Jones J, Ritchie KB, Jones LE, Ellner SP (2010) How Microbial Community Composition Regulates Coral Disease Development. doi:10.1371/journal.pbio.1000345**

**Figure:**
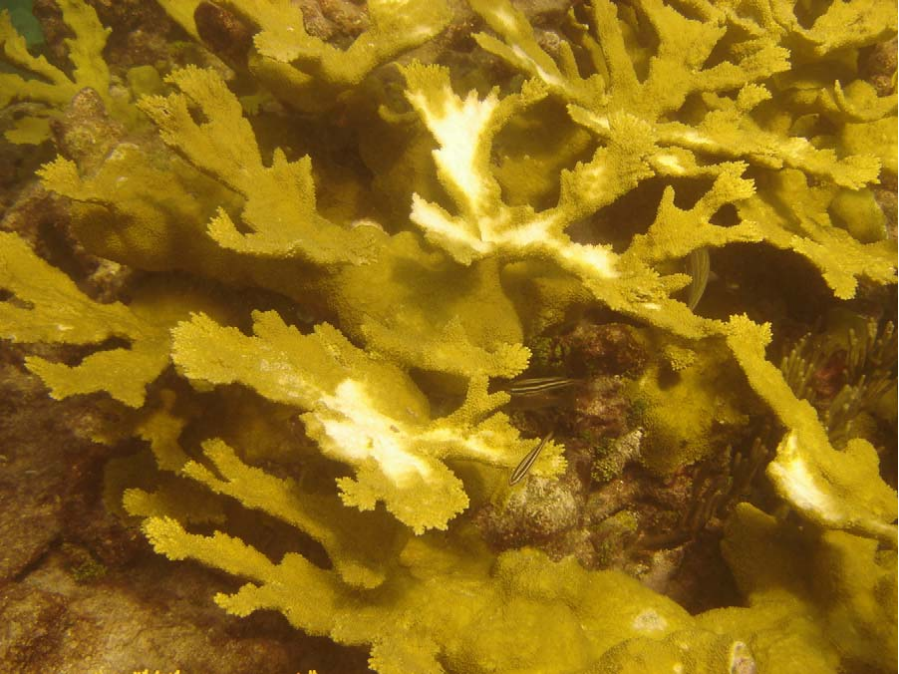
*Acropora palmata* coral colonies displaying partial bleaching (Looe Key Reef, Florida Keys National Marine Sanctuary). Image: Erich Bartels, Mote Marine Laboratory.

